# From dusk till dawn: the *Arabidopsis thaliana* sugar starving responsive network

**DOI:** 10.3389/fpls.2014.00482

**Published:** 2014-09-22

**Authors:** Maria C. Arias, Sandra Pelletier, Frédérique Hilliou, Fabrice Wattebled, Jean-Pierre Renou, Christophe D'Hulst

**Affiliations:** ^1^Unité Glycobiologie Structurale et Fonctionnelle, UMR 8576-CNRS, Université de Lille 1Villeneuve d'Ascq, France; ^2^URGV, UMR INRA 1165-CNRS 8114-UEVEÉvry, France; ^3^Institut Sophia Agrobiotech, UMR 1355, Institut National de la Recherche AgronomiqueSophia-Antipolis, France

**Keywords:** co-expression network analysis, starch, sugar starvation, microarrays, metabolism integration

## Abstract

Plant growth and development are tightly controlled by photosynthetic carbon availability. The understanding of mechanisms governing carbon partitioning in plants will be a valuable tool in order to satisfy the rising global demand for food and biofuel. The goal of this study was to determine if sugar starvation responses were transcriptionally coordinated in *Arabidopsis thaliana*. A set of sugar-starvation responsive (SSR) genes was selected to perform a co-expression network analysis. Posteriorly, a guided-gene approach was used to identify the SSR-network from public data and to discover candidate regulators of this network. In order to validate the SSR network, a global transcriptome analysis was realized on three *A. thaliana* starch-deficient mutants. The starch-deficient phenotype in leaves induces sugar starvation syndrome at the end of the night due to the absence of photosynthesis. Promoter sequences of genes belonging to the SSR-network were analyzed *in silico* reveling over-represented motifs implicated in light, abscisic acid, and sugar responses. A small cluster of protein encoding genes belonging to different metabolic pathways, including three regulatory proteins, a protein kinase, a transcription factor, and a blue light receptor, were identified as the cornerstones of the SSR co-expression network. In summary, a large transcriptionally coordinated SSR network was identified and was validated with transcriptional data from three starch-deficient mutant lines. Candidate master regulators of this network were point out.

## Introduction

The photosynthetic carbon fixation and the subsequent synthesis of polysaccharides such a starch and cellulose are necessary to plant development and growth, but they also play a vital role for life on earth. Biosynthesis, transport, and storage of carbohydrates depend on plant physiology but also on environmental conditions, and the capacity of plants to sense and respond to sugars is a pivotal element between internal and external signals (Koch, 2004; Rolland et al., 2006; Loreti et al., 2008; Lastdrager et al., 2014). It is expected that plant growth is driven by carbohydrates availability, but the existence of more complex regulatory mechanisms coordinating carbon supply and use have been proposed (Smith and Stitt, 2007). It has been suggested that starch synthesis and degradation is a crucial element in the balance between resource availability and development in leaves (Sulpice et al., 2009). Starch is a primary product of photosynthesis, and the most important storage-carbohydrate in plants (Buleon et al., 1998); its metabolism is complex and involves several enzymes encoded by genetically independent nuclear-genes (Ball and Morell, 2003). Although the understanding of the molecular mechanisms that lead to starch metabolism in leaves has been strongly improved in the last decade, the integration of the starch biosynthetic pathway with the whole plant carbon metabolism has not been fully clarified (Kotting et al., 2010). However, evidence showing the articulation of starch metabolism with other primary pathways has been shown (Mentzen et al., 2008).

During the last few years, the study of plant gene networks has become possible thanks to the huge available amount of data generated by *Arabidopsis thaliana* knock-out mutants and from plants undergoing multiple physiological conditions (e.g., hormonal and chemical treatments; biotic or abiotic stresses). Network representation of transcriptomic data can provide evidence of gene-to-gene relationships. Network analysis may uncover groups (modules) of functionally related genes and thus, can yield a systematic understanding of plant responses. The classic clustering analysis is a useful approach to identify genes that may participate in the same biological process (D'Haeseleer, 2005), but is not efficient for the identification of regulatory genes. By revealing biological relationships that are not apparent from single gene co-expression approaches, co-expression network analysis is a powerful integrative approach for the construction of new data-driven models (Barabasi and Oltvai, 2004).

In the present work a guided-gene approach was chosen to study the sugar starvation syndrome at transcriptional level (Lisso et al., 2005; Aoki et al., 2007). A small group of sugar starving responsive genes (SSR) were selected, to perform a co-expression network analysis. This gene subset was loaded into the CressExpress pipeline, a co-expression analysis tool for *A. thaliana* microarray expression data (Srinivasasainagendra et al., 2008), and Cytoscape software package (Cline et al., 2007) was posteriorly used to build a graphical representation of the network.

The SSR network was validated with transcriptional data from starch-impaired mutants. In order to mimic sugar starvation in leaves, microarray analysis was performed on three *A. thaliana* starch-deficient mutants obtained in our lab (Dumez et al., 2006). The starchless mutant *be2 be3*, knock-out of two genetically independent isoforms of starch branching enzyme (Dumez et al., 2006), was compared with an *adg1* insertion mutant line, with the leaves starch content reduced by 80% at the end of the day. The *ADG1* gene encodes the small catalytic subunit of ADPG-pyrophosphorylase. This enzyme is responsible for the synthesis of ADP-glucose, the unique precursor of starch synthesis. In the double mutant *be2 be3* starch synthesis was abolish, substituted by abnormally high levels of maltose in the cytosol, likely due to the incapacity of cells to integrate it into the cellular metabolism. However, maltose accumulation disappeared when the *adg1* mutant allele was combined with both *be2* and *be3* mutations to generate the triple mutant *adg1 be2 be3* (Dumez et al., 2006). This triple mutant was also included in our analysis.

## Materials and methods

### SSR genes selection and co-expression network analysis

According to the hypothesis that genes involved in coordinated biological processes share similar transcription profiles (Eisen et al., 1998), 13 sugar-starvation responsive genes were selected to perform a co-expression network analysis (Kolbe et al., 2005; Gonzali et al., 2006; Binder et al., 2007; Lee et al., 2007; Hanson et al., 2008; Miyashita and Good, 2008). Genes of this subset belong to few metabolic pathways (Table 1). In order to enlarge this first network, two more correlation coefficient analysis were performed in a recursive way, including significantly co-expressed genes with at least 5 neighbors from each previous analysis. CressExpress pipeline (http://www.cressexpress.org/index.jsp) was used for the construction of the co-expression network. CressExpress computes patterns of correlated expression between query genes and the rest of the genes in the genome (Srinivasasainagendra et al., 2008). The co-variation analysis was carried out on the data release 3.0 with all available samples, and with CressExpress default settings. Finally, Cytoscape software package (http://www.cytoscape.org/) was used to perform a graphical representation of the network (Cline et al., 2007). Highly interconnected modules were identified by AllegroMCODE (http://allegroviva.com/allegromcode/), a high-performance MCODE cluster finder for Cytoscape (Saito et al., 2012). All genes with at least two neighbors were represented in the final SSR network.

**Table 1:** **SSR gene subset used as seeds for the recursive expression network analysis**.

**Pathway**	**AGI code**	**Gene name**	**TAIR annotation**	**References**
Proline catabolism	At3g30775	ERD5	Proline dehydrogenase	Hanson et al., 2008
Asparagine metabolism	At3g47340	ASN1	Asparagine synthetase	Hanson et al., 2008
	At3g45300	IVD	Isovaleryl-CoA-dehydrogenase	Binder et al., 2007
BCAA degradation	At3g13450	BCKDH E1β	BC α-keto acid dehydrogenase	Binder et al., 2007
	At3g06850	BCKDH E2	BC α-keto acid dehydrogenase	Binder et al., 2007
	At1g03090	MCCA sub1	3-methylcrotonyl-CoA carboxylase	Binder et al., 2007
	At4g34030	MCCB sub2	3-methylcrotonyl-CoA carboxylase	Binder et al., 2007
Glutamate metabolism	At5g07440	GDH2	Glutamate dehydrogenase 2	Miyashita and Good, 2008
Cell wall turnover	At5g56870	BGAL4	β-galactosidase	Lee et al., 2007
	At5g49360	BXL1	Glycosyl hydrolase family 3	Lee et al., 2007
Trehalose biosynthesis	At2g18700	TPS11	Trehalose-6-phosphate synthase	Kolbe et al., 2005
	At1g70290	TPS8	Trehalose-6-phosphate synthase	Kolbe et al., 2005
Dormancy	At1g28330	DRM1	Dormancy-associated protein	Gonzali et al., 2006

### *In silico* analysis of cis-regulatory elements

ATHENA (http://www.bioinformatics2.wsu.edu/Athena) promoter sequence analysis tool (O'Connor et al., 2005) was used to identify statistically over-represented (*p* < 10^−3^) transcription factor binding sites occurring in the selected set of promoters (1000 bp upstream of the transcriptional start site). The PROMOMER tool (http://bar.utoronto.ca/ntools/cgi-bin/BAR_Promomer.cgi) was also used to identify statistically over-represented cis-elements in the promoters of the SSR genes (Toufighi et al., 2005). PROMOMER is a word-counting program that allows the identification of significantly over-represented motifs in a particular set of promoters compared with randomly generated promoter sets from the genome (Toufighi et al., 2005). This tool uses the Boyer–Moore algorithm for matching and counting n-mers in a set of sequences (Boyer and Moore, 1977).

### *Arabidopsis thaliana* lines and growth conditions

Three *A. thaliana* mutant lines ecotype Wassilewskija were used in this study: the single mutant *adg1*, the double mutant *be2 be3* and the triple mutant *adg1 be2 be3*. The double and triple mutant were generated in our lab (Dumez et al., 2006). In *adg1* the T-DNA was inserted in the promoter region, leading to a non-null phenotype. This mutant line presents a reduction of 80% of the leaf starch content at the end of the day. The conditions in the environmental chamber were as follows: 100 μE.m^−2^.s^−1^ irradiance, 16/8 light/dark cycle, 23/20°C. Leaf samples were harvested on 6-week-old plants.

### Transcriptomic studies

Microarray analysis was carried out using the CATMA arrays containing 24,576 gene-specific tags corresponding to 22,089 genes from Arabidopsis (Crowe et al., 2003; Hilson et al., 2004). Each mutant line was compared with control plants. Three independent biological replicates were produced. For each biological repetition, leaves of three plants were randomly selected and immediately frozen in liquid nitrogen. Leaves were collected at two time points: at the end of the light phase (qualified hereafter as “dusk”) and at the end of the dark phase (qualified hereafter as “dawn”). Samples were collected on plants at 5.10 developmental growth stage (Boyes et al., 2001). Total RNA was extracted using the Plant RNeasy kit (QIAGEN, Courtaboeuf, France) according to the supplier's instructions. For each comparison, one technical replication with fluorochrome dye swap was performed for each biological replicate (i.e., four hybridizations per comparison). The labeling of cRNAs with Cy3-dUTP or Cy5-dUTP (Perkin-Elmer-NEN Life Science Products), the hybridization to the slides, and the scanning were performed as described in Lurin et al. (2004).

### Statistical analysis of microarray data

Normalization and statistical analysis was based on three dye swaps (i.e., six arrays, each containing 24,576 GSTs and 384 controls) as described in Gagnot et al. (2008). To determine differentially expressed genes, we performed a paired *t*-test on the log ratios, assuming that the variance of the log ratios was the same for all genes. Spots displaying extreme variance (too small or too large) were excluded. The raw *p*-values were adjusted by the Bonferroni method (Ge et al., 2003). We considered as being differentially expressed the genes with a Bonferroni *p* ≤ 0.05, as described in Gagnot et al. (2008). Microarray data were deposited at Gene Expression Omnibus (http://www.ncbi.nlm.nih.gov/geo/), accession number GSE20964, and at CATdb (http://urgv.evry.inra.fr/CATdb), project: GnP06-02_Starch; according to the “Minimum Information about a Microarray Experiment” standards.

## Results

### Co-expression network analysis

**Figure 1:**
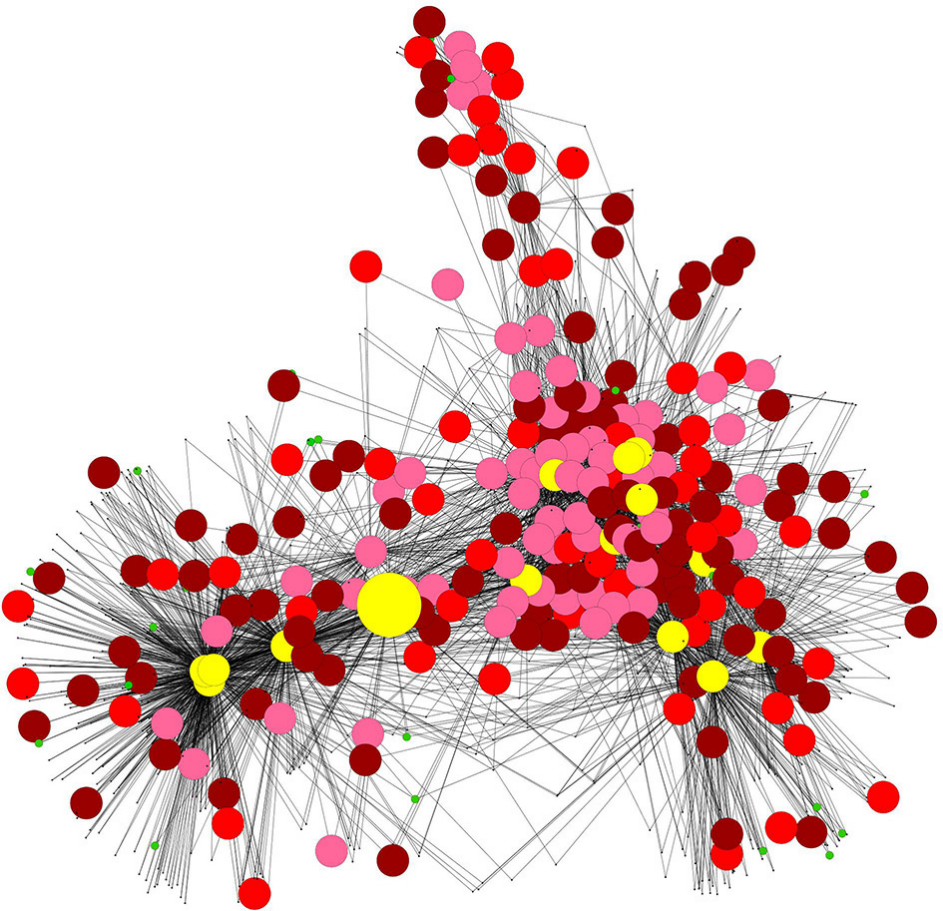
**SSR network**. Co-expression network analysis performed with CressExpress using public transcriptome datasets. Cytoscape was used for the graphical representation of the network. Pink circles, red circles and purple circles indicate respectively genes induced at dawn in three, two or only one of the starch-deficient mutant lines. Green circles indicate genes repressed at the end of the night. Black dots indicate genes with stable expression in the three mutant lines under the conditions of the present study. The yellow circles correspond to the 15 genes highly connected (more than 100 neighbors). These candidate regulatory genes belong to several metabolic pathways: lipid catabolism (*At3g51840, At5g04040*), cell redox homeostasis regulation (*At2g37130, At5g10860*), amino acid metabolism (*At2g39570, At2g40420, At3g45300*), protein transport (*At1g80920*), trehalose biosynthesis (*At1g23870*), three uncharacterized proteins (*At4g28300, At4g28040, At2g32150*), a protein kinase (*STY46*), a transcription factor (*UPBEAT1*) and a blue light receptor (*PLPB*). The central bigger yellow circle correspond to the protein kinase *STY46*.

A SSR network constituted by 820 nodes (genes) and 3880 edges was obtained (Figure 1) using the selected 13 genes as seeds (Table 1). The SSR network have the classical scale-free topology of biological networks, showing few highly connected nodes interacting with many poorly connected nodes (Barabasi and Oltvai, 2004). The large majority of genes in the network (717) have less than 10 neighbors, 11% of the genes (89) have between 10 and 99 neighbors; only 2% of the genes (15) have more than 100 neighbors, and could be considered as potential master regulators. These 15 candidate master regulators belong to several metabolic pathways: lipid catabolism (*At3g51840, At5g04040*), cell redox homeostasis regulation (*At2g37130, At5g10860*), amino acid metabolism (*At2g39570, At2g40420, At3g45300*), protein transport (*At1g80920*), trehalose biosynthesis (*At1g23870*), and three uncharacterized proteins (*At4g28300, At4g28040, At2g32150*). Interestingly, three of the highly connected genes encode for plant-specific regulatory proteins with unknown metabolic function: a protein kinase (*STY46*), a transcription factor (*UPBEAT1*) and a blue light receptor (*PLPB*). All these regulator genes were overexpressed in the starch-deficient mutant lines (Supplemental Data Sheet 1). The gene encoding for the isovaleryl-CoA-dehydrogenase (*IVD, At3g45300*) is the only candidate regulatory gene that belongs to the starting SSR gene set. The SSR network was searched for modules, defined as a group of densely connected nodes (genes) that have a sparsely connected periphery. Two highly interconnected modules were clearly identified by AllegroMCODE (Supplemental Image 1). Remarkably, the protein kinase *STY46* tightly connects these two modules (Supplemental Data Sheet 1). Several of *STY46* neighbors are in turn densely connected, suggesting a central role of this gene in the SSR network (Figure 1).

### Starch-deficient mutants

**Figure 2:**
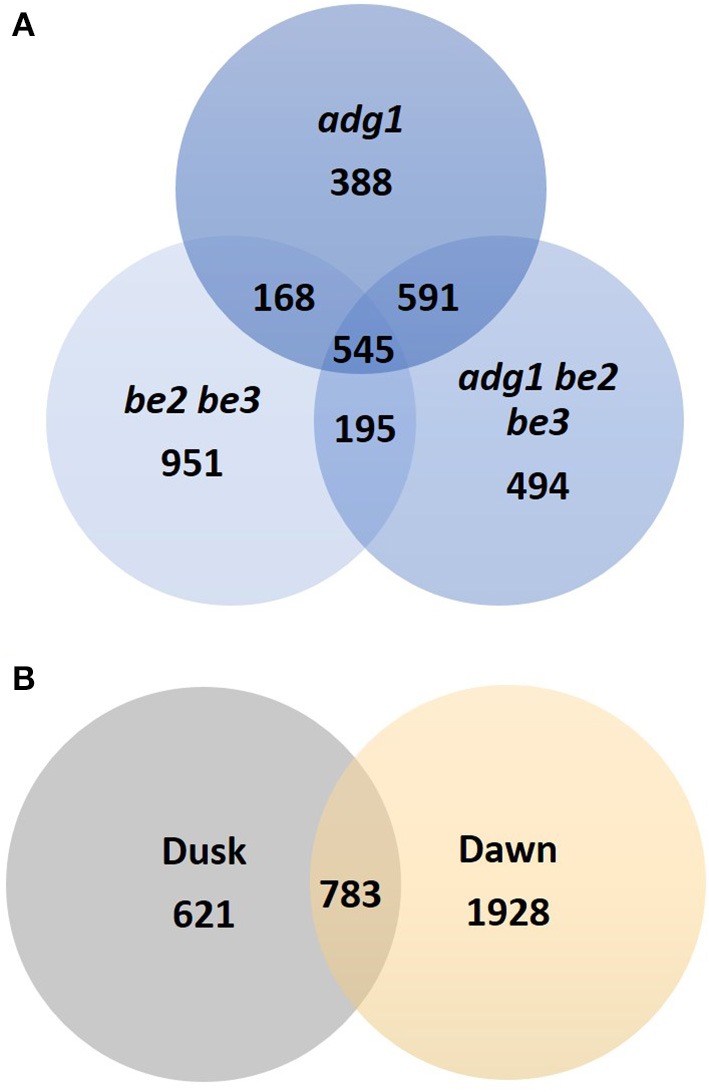
**Venn diagrams of genes identified as differentially expressed. (A)** Genes identified as differentially expressed in *adg1, adg1 be2 be3*, and *be2 be3* mutants. **(B)** Venn diagram showing the circadian regulation of differentially expressed genes in the three mutant lines.

The diurnal cycle provides a defined experimental system to investigate how the supply and utilization of carbon is coordinated within the plant tissues. In wild-type plants leaves, starch is synthesized during the day, while photosynthesis is active, and degraded during the night providing a continuous supply of carbon and energy to sustain plant metabolism and growth. 3332 genes displayed a significantly altered expression pattern in at least one of the mutants (Bonferroni *p* < 0.05) and 545 of them were modulated simultaneously in the three mutant lines (Figure 2A). Samples were taken at two time points between light and dark phase: “dusk” and “dawn.” The global response during the diurnal cycle showed a large reprogramming of the cell transcriptome in the three starch-deficient mutants at the end of the dark phase (“dawn”). Only 23% of genes (783) showed an altered expression pattern at both times of sampling, but the majority, 77% (2549), were modulated either at dawn or at dusk, strongly suggesting a circadian-regulated expression (Figure 2B).

**Figure 3:**
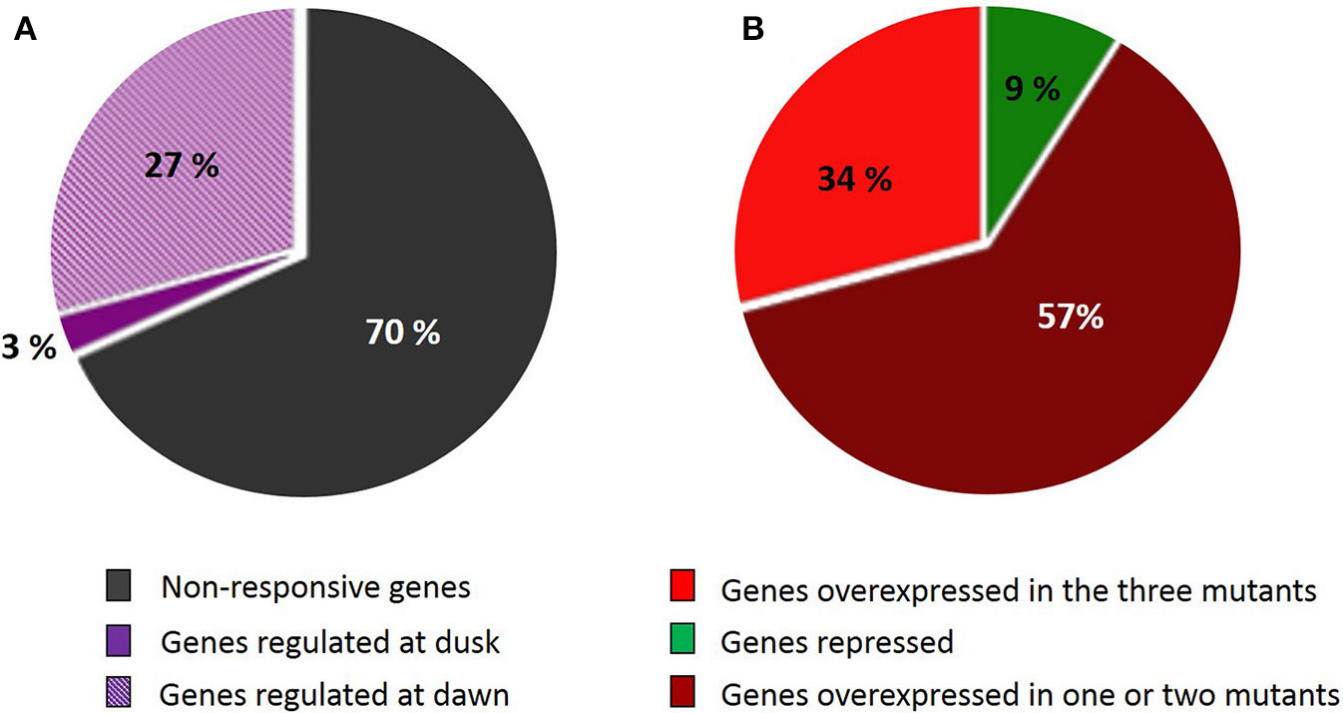
**Genes belonging to the SSR network showing different pattern of expression in the starch-deficient mutant lines**. **(A)** The ripped zone represents the percentage of genes repressed or overexpressed at dawn in at least one of the mutants; the violet zone represents genes up- or down-regulated at dusk in at least one of the mutants and the gray zone represents genes showing a stable level of expression at the two times of sampling. **(B)** Percentage of genes that repressed or overexpressed in *adg1, adg1 be2 be3*, and *be2 be3* microarray experiments at dawn (ripped zone of **A**). Red color represents genes overexpressed in the three mutants; dark red represents genes overexpressed in one or two mutant lines and green represents genes repressed in the starch-deficient mutant lines.

Microarrays data from the three starch-deficient mutant lines were cross-examined with the SSR gene network. Two hundred and twenty genes of the network (27%) (Figure 3A) were overexpressed at dawn in at least one of the mutants (Supplemental Data Sheet 1), and 74 of these genes were overexpressed in the three mutant lines (Figure 3B). At this time point, starch-deficient plants suffer from sugar starvation as a consequence of the absence of photosynthesis during the dark phase. Interestingly, the expression of SSR genes were nearly not modified at dusk, when plants are not under starvation. Only 27 genes of the network were underexpressed at dawn, but none of them was repressed simultaneously in the three mutant lines. Remarkably, all the genes of the SSR network having more than 25 neighbors were overexpressed at dawn in *adg1, be2 be3*, and/or *adg1 be2 be3* (Supplemental Data Sheet1). On the contrary, starchless non-responsive genes were located mostly in the periphery of the network (Figure 1). None of these genes interacted with more than 25 neighbors, indicating that they were not implicated in the structure and coordination of the SSR network.

### Promoter analysis

An *in-silico* analysis was performed on the promoter sequences of the SSR genes in order to identify cis-elements possibly implicated in the regulation of the transcriptional network. ATHENA pipeline was used to search known transcription factor binding sites in the 220 genes overexpressed in the starch-deficient mutant lines (O'Connor et al., 2005). Two cis-elements were significantly over-represented (Table 2): Ibox promoter motif, a conserved motif upstream of light regulated genes (Giuliano et al., 1988), and ABRE-like binding site, a family of abscisic acid responsive elements (Abe et al., 2003; O'Connor et al., 2005). Posteriorly, a non-targeted approach was performed using PROMOMER (Toufighi et al., 2005). TTATC, a fragment of the sugar repressive element TTATCC (Tatematsu et al., 2005), was found in 85% of promoters (Supplemental Data Sheet 2). Remarkably, GATAA, an Ibox associated motif (Giuliano et al., 1988), was also significantly over-represented in the SSR genes (86%), in coherence with the results obtained with ATHENA (Supplemental Data Sheet 3).

**Table 2:** **Transcription factor-binding sites**.

**Transcription factor motif name**	**Cis-elements in subset (220 genes)**	**Cis-elements in genome**	***p*-value**
ABRE-like binding site	39% 89	20% 6258	<10e^−5^
Ibox	62% 140	40% 12259	<10e^−4^

*Most common cis-acting promoter elements within the 200 SSR genes overexpressed in the starch deficient mutant lines identified by ATHENA pipeline*.

### SSR metabolic pathways

The subset of SSR genes selected for the co-expression network analysis was involved in amino acid metabolism, cell wall turnover, trehalose biosynthesis, and dormancy (Table 1). Remarkably, the resulting SSR network included also pathways that were not present in the starting gene set. Genes with the highest number of neighbors were involved in amino acid metabolism, cell wall metabolism, lipid catabolism, sugar signaling, transcription regulation, and cell redox homeostasis. Genes related with metabolites transport, proteolysis, detoxification and stress response, signal transduction, and several genes with unknown function were also part of the network (Supplemental Data Sheet 1).

#### Cell wall metabolism

A small cluster of genes involved in cell wall metabolism including the sugar starving responsive *BGAL4* and *BXL1* (Lee et al., 2007), were part of the SSR network and were also overexpressed at the end of the dark phase in the starch-deficient mutant lines (Supplemental Data Sheet 4). Cell wall metabolism is connected with the central plant metabolism through the nucleotide sugar interconversion pathway (Seifert, 2004). *UGE1*, coding for an enzyme that interconverts UDP-galactose to UDP-glucose, was part of the SSR network and was activated at dawn in the triple mutant *adg1 be2 be3*. UDP-glucose, through the generation of glucose-6-P, is a precursor for both fatty acid and trehalose synthesis (Avonce et al., 2006; Barber et al., 2006). Interestingly, three trehalose-phosphate synthases (Supplemental Data Sheet 4), and several genes involved in lipid degradation were part of the SSR network and were also activated at dawn in the mutant lines (Supplemental Data Sheet 5).

The three starch-deficient mutants showed an altered expression pattern of cell wall metabolic genes. A large cluster of genes implicated in the biosynthesis of cell wall polysaccharides, but also in cell wall construction and modification were repressed in *adg1, adg1 be2 be3*, and *be2 be3* mutant lines at the end of the dark phase (Supplemental Data Sheet 4).

#### Sugar signaling

SnRK1-like proteins (Sucrose non-Fermenting Related Kinase 1) have been implicated in the regulation of energy and stress signal transduction pathways in eukaryotes (Hey et al., 2010). Trehalose-6-phosphate (T6P) is a plant signaling sugar that inhibits SnRK1 and is synthesized by the trehalose biosynthetic pathway. Several genes related with the T6P/SnRK1 signaling pathway were part of the SSR network. In particular *KINβ1* (*At5g21170*), *KING1* (*At3g48530*), and *ATHSPRO2* (*At2g40000*), but also five trehalose-phosphate synthases, *TPS6, TPS8, TPS9, TPS10, TPS11*. All of these genes with the exception of *TPS6* and *TPS10* were overexpressed at dawn in the starch-deficient mutant lines (Supplemental Data Sheet 4).

#### Lipid metabolism

Interestingly, despite being absent of the starting SSR gene subset, genes involved in lipid catabolism were central elements of the SSR network, and they were also overexpressed at the end of the dark phase in the mutants (Supplemental Data Sheet 5). In particular two candidate master regulators: *ACX4*, a short-chain acyl-CoA oxidase involved in fatty acid β-oxidation, and *SDP*, an oil body triacylglycerol lipase (Figure 1, Supplemental Data Sheet 1). An oleosin (*At5g56100*) encoding for a protein present in oil bodies, a peroxin (*At3g61070*) member of a gene family that controls peroxisome proliferation, an acyl-CoA oxidase (*ACX1*) involved in fatty acid degradation, and two lipases (*At1g02660, At3g62860*) were also overexpressed in the starch-deficient mutant lines, but were not part of the SSR transcriptional network. On the contrary, cuticle biosynthesis-related genes, a secondary pathway associated with lipid metabolism, were largely repressed at dawn in the starch-deficient mutant lines (Supplemental Data Sheet 5).

#### Amino acid metabolism

Several genes involved in the amino acids metabolism and turnover were part of the SSR network, and were also overexpressed at the end of the dark phase in the three mutant lines (Supplemental Data Sheet 1). In particular, *BCKDH E1β, BCKDH E2, IVD, MCCA, MCCB*, and *DIN4* involved in the degradation of branched chain amino acids (BCAA) (Supplemental Data Sheet 7). Acetolactate synthase the first committed step of BCAA biosynthesis in plants and bacteria (Duggleby et al., 2008). The acetolactate synthase small subunit coding gene (*AHASS1*), was also overexpressed in the starch-deficient mutant lines (Supplemental Data Sheet 6). *AHASS1* was not part of the SSR transcriptional network indicating a differential regulation of this gene (Gao et al., 2014). BCAA catabolism is induced in *A. thaliana* under sugar starvation (Binder et al., 2007); however, *IMS2* and *IMS3*, were repressed in the mutant lines (Supplemental Data Sheet 7). These two genes encode enzymes shared by the methionine and the BCAA biosynthetic pathway, and are involved in the synthesis of glucosinolate precursors (Binder et al., 2007). We observed several secondary metabolic pathways displaying an altered expression pattern on the three starch-deficient mutants. Genes involved in the synthesis of glucosinolates (Supplemental Data Sheet 6), lignin, polyamines, phenylpropanoids and flavonoids were repressed at the end of the dark phase (Supplemental Data Sheet 7) suggesting a large-scale negative regulation of the secondary metabolism in the starch-deficient mutant lines.

## Discussion

Carbon availability and light are two important cross-talking signals regulating plant growth and development (Fankhauser and Chory, 1997; Moore et al., 2003; Casal and Yanovsky, 2005; Franklin et al., 2005; Rolland et al., 2006). They modulate gene expression through signal transduction cascades affecting downstream cellular responses (Gibson, 2005; Osuna et al., 2007; Thum et al., 2008). The goal of the present study was to analyze the sugar starvation syndrome at transcriptional level. Although some data suggest the articulation of lipid, amino acid and starch pathways (Mentzen et al., 2008), evidences of a large-scale coordinated plant response to sugar starving are still lacking. A subset of 13 SSR genes (Table 1) were used as seeds for the co-expression analysis. The resulting SSR network was composed by 820 genes involved in amino acid metabolism, cell wall biosynthesis, and sugar signaling, but also by pathways not present in the starting gene set such as lipid metabolism and transcription regulation. A small cluster of 15 genes were identified as the cornerstones of the SSR network, in particular *STY46*, a protein kinase encoding gene. *STY46* is co-expressed with most of the genes responsible of the network structure. The protein kinase *STY46* has been implicated in the phosphorylation of proteins targeted to chloroplast (Martin et al., 2006), but its metabolic function is still unknown.

The SSR network was validated with transcriptional data from three starch-deficient mutant lines obtained in our lab (Dumez et al., 2006). Two hundred and twenty genes of the SSR network were overexpressed in our mutant lines at dawn. The presence of a residual amount of starch in *adg1* at the end of the night was not enough to prevent the activation of SSR genes, in coherence with previous data (Graf et al., 2010; Scialdone et al., 2013). This result also confirms that maltose accumulation in *be2 be3* cannot overcome the starch depletion (Dumez et al., 2006). Cis-elements responsive to light, abscisic acid, and sugars were over-represented in the promoter regions of the SSR genes, strongly suggesting that their expression is transcriptionally coordinated.

The diurnal cycle controls gene expression and plant metabolism (Blasing et al., 2005). In coherence with previous studies, 75% of responsive genes display a circadian expression pattern in *adg1, be2 be3*, and *adg1 be2 be3*. Our data showed that sugar starvation caused by starch deficiency induce a generalized transcriptional reprogramming in the three mutants at the end of the night. Plant nitrogen-nutrition is tightly linked to the circadian rhythm (Gutierez et al., 2008). It is known that sugars can regulate amino acid metabolic genes in plants (Silvente et al., 2008), and that sugar starvation induces amino acid biosynthesis (Mentzen et al., 2008; Miyashita and Good, 2008). Remarkably, amino acid metabolic genes were central elements in the organization of the SSR network and were also overexpressed in the starch-deficient mutant lines (Supplemental Data Sheet 1). Amino acids are indispensable for protein synthesis, but also for other metabolic pathways because they are precursors of a large number of primary and secondary metabolites (D'Auria and Gershenzon, 2005). In particular, they are indispensable for the lignin biosynthesis, an essential constituent of the cell walls, *via* the flavonoid and phenylpropanoid biosynthetic pathway (Besseau et al., 2007).

Plant cell wall is a complex and composite material composed predominantly of polysaccharide networks consisting of cellulose, hemicelluloses and pectin, with an external hydrophobic cover, the cuticle (Lerouxel et al., 2006; Pollard et al., 2008). A small cluster of genes involved in cell wall metabolism was part of the SSR network, and was also activated in the starch-deficient mutant lines. It has been shown that the inhibition of photosynthesis induce the expression of *BGAL4* and *BXL1* genes and the corresponding protein accumulation (Lee et al., 2007). These data suggest that cell wall may function as a reserve of carbon under severe sugar starving conditions, such as those found in starch-deficient mutants. In agreement with this hypothesis, cell wall metabolic genes were largely repressed at the end of the night in the mutants used in this work (Supplemental Data Sheet 4), when plants suffer sugar starvation due to the absence of photosynthesis.

Sugars are important signaling molecules for the coordination of plant metabolism (Rolland and Sheen, 2005). Five trehalose-phosphate synthases coding genes were part of the SSR network. Trehalose biosynthetic pathways are implicated in the crosstalk between central metabolism and development in several species (Paul et al., 2008). T6P, the trehalose precursor, is mandatory for the coordination of carbon utilization and plant growth, regulating starch synthesis in plastids of higher plants (Eastmond and Graham, 2003). T6P is synthesized in the cytosol and acts on plastidial metabolism by promoting thioredoxin-mediated redox transfer to AGPase in response to cytosolic sugar levels, thereby allowing starch synthesis to be regulated independently of light. It has been shown that over-expression of T6P synthase increase T6P levels (Kolbe et al., 2005). We speculate that this type of mechanism could be induced in the starch-deficient mutant lines. T6P is also implicated in sugar signaling, altering downstream gene expression through the inhibition of SnRK1 (SNF1-related protein kinase). It has been proposed that the T6P/SnRK1 signaling pathway responds to sugar starvation enabling growth recovery under sink restriction (Nunes et al., 2013). Remarkably, three genes involved in SnRK1 signaling pathway were also part of the SSR network.

**Figure 4:**
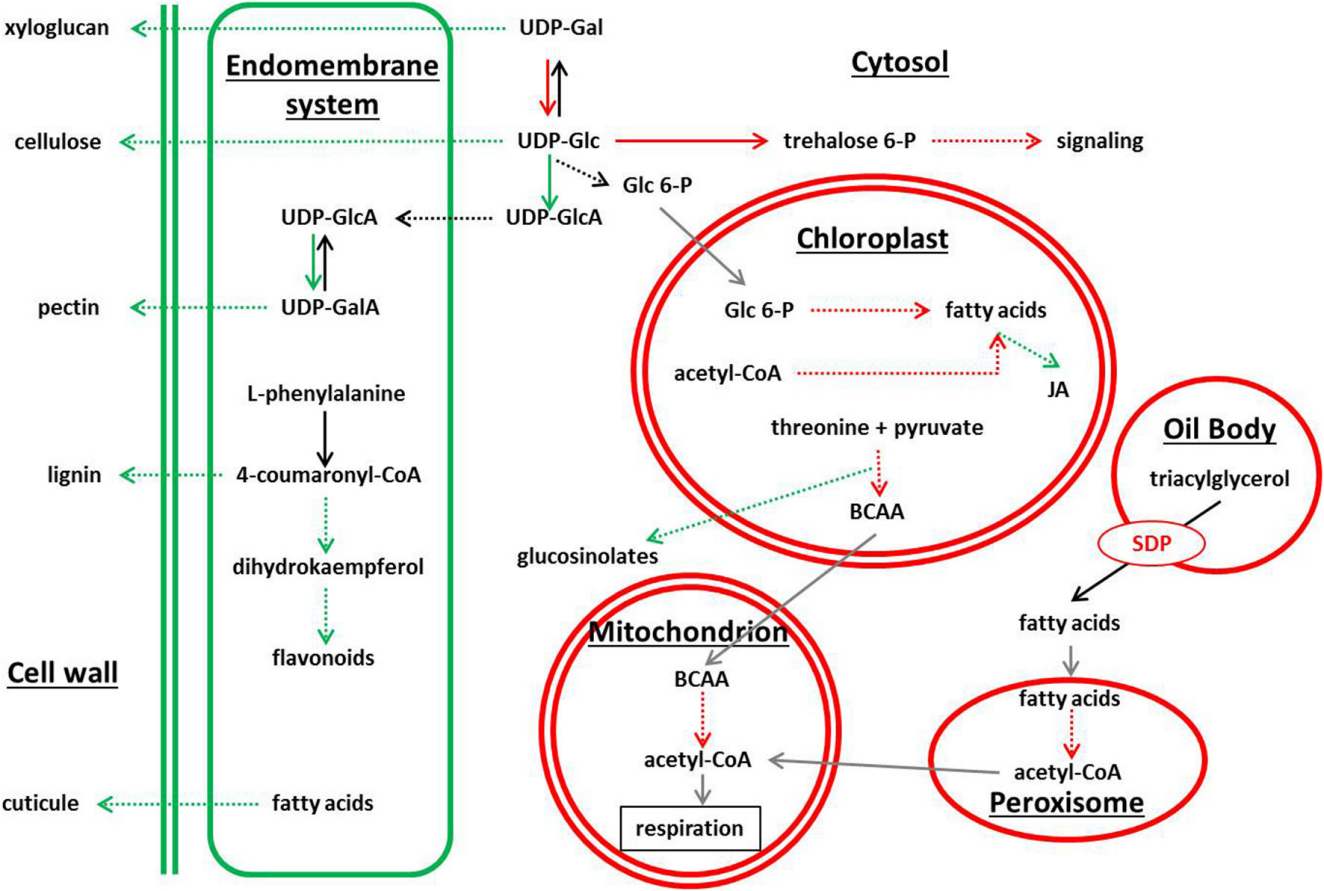
**Schematic view of metabolic pathways showing differential gene expression in *adg1, adg1 be2 be3*, and *be2 be3* mutants**. Green color and red color correspond to pathways showing repressed genes and overexpressed genes, respectively.

Genes involved on secondary pathways dependent of amino acids and lipids, such us cuticle glucosinolate, flavonoids, and phenylpropanoids metabolism (Baker et al., 2006; Pollard et al., 2008), were largely repressed in the starch-deficient mutants. Intriguingly, the chalcone-flavanone isomerase-like coding gene (*At5g05270*) was strongly repressed in the three mutant lines (Supplemental Data Sheet 7). This enzyme with unknown function is the evolutionary connection between fatty acid and flavonoid metabolism (Ngaki et al., 2012), and our results suggest its implication in the sugar starvation syndrome. On the other hand, genes related with lipid metabolism were overexpressed in *adg1, adg1 be2 be3*, and *be2 be3*, and were highly connected in the SSR network. Surprisingly, *SD1* a triacylglycerol hydrolase normally expressed in seed oil bodies, and *ACX4* a short-chain acyl-CoA oxidase involved in fatty acid β-oxidation that takes place mostly during seed germination (Graham, 2008), were identified as possible master regulators of the network. Other genes normally expressed in seeds and seedlings were also highly overexpressed in the three mutant lines: an oleosin present in oil bodies (*At5g56100*), a peroxin (*At3g61070*) member of a gene family that controls peroxisome proliferation, where occurs fatty acid degradation (Poirier et al., 2006), and also several genes involved in fatty acid β-oxidation. Interestingly, triacylglycerol hydrolysis plays a pivotal role in life cycle by providing the carbon skeletons and energy that drive post-germination growth in several plants species (Eastmond, 2006). At seedling stage, lipid resources are mobilized to provide carbon and energy to sustain plants growth. The over-expression of these genes in the starch-deficient mutants is puzzling, suggesting that a metabolic switch from starch metabolism to lipid metabolism take place in these plants (Figure 4).

## Conclusions

It is generally accepted that plant growth is dependent on carbohydrates availability. Plants are capable to adjust the rate of starch degradation during the night in order to avoid carbon starvation, but in starch-deficient mutants this mechanism is not possible. Our data strongly suggest that under sugar starving conditions, plants can reallocate their metabolism to limit carbon starvation in a response transcriptionally coordinated. Less mandatory pathways such as cell wall biosynthesis and secondary metabolites biosynthesis are neglected as far as they don't compromise plant survival and growth. Our data-driven co-expression network analysis has enlightened candidate regulators of the SSR network, in particular embryo-induced genes, usually not expressed in leaves. The overexpression of lipid metabolism embryo-induced genes at the end of the night in starch-deficient mutant lines strongly suggest a metabolic shift from autotrophy to heterotrophy in response to sugar starvation.

### Conflict of interest statement

The authors declare that the research was conducted in the absence of any commercial or financial relationships that could be construed as a potential conflict of interest.
