# Supplementary figures and images for: From dusk till dawn: the Arabidopsis thaliana sugar starving responsive network
Source: Front Plant Sci. 2014 Sep 22;5:482. doi: 10.3389/fpls.2014.00482 (PMC4170100; doi:10.3389/fpls.2014.00482)

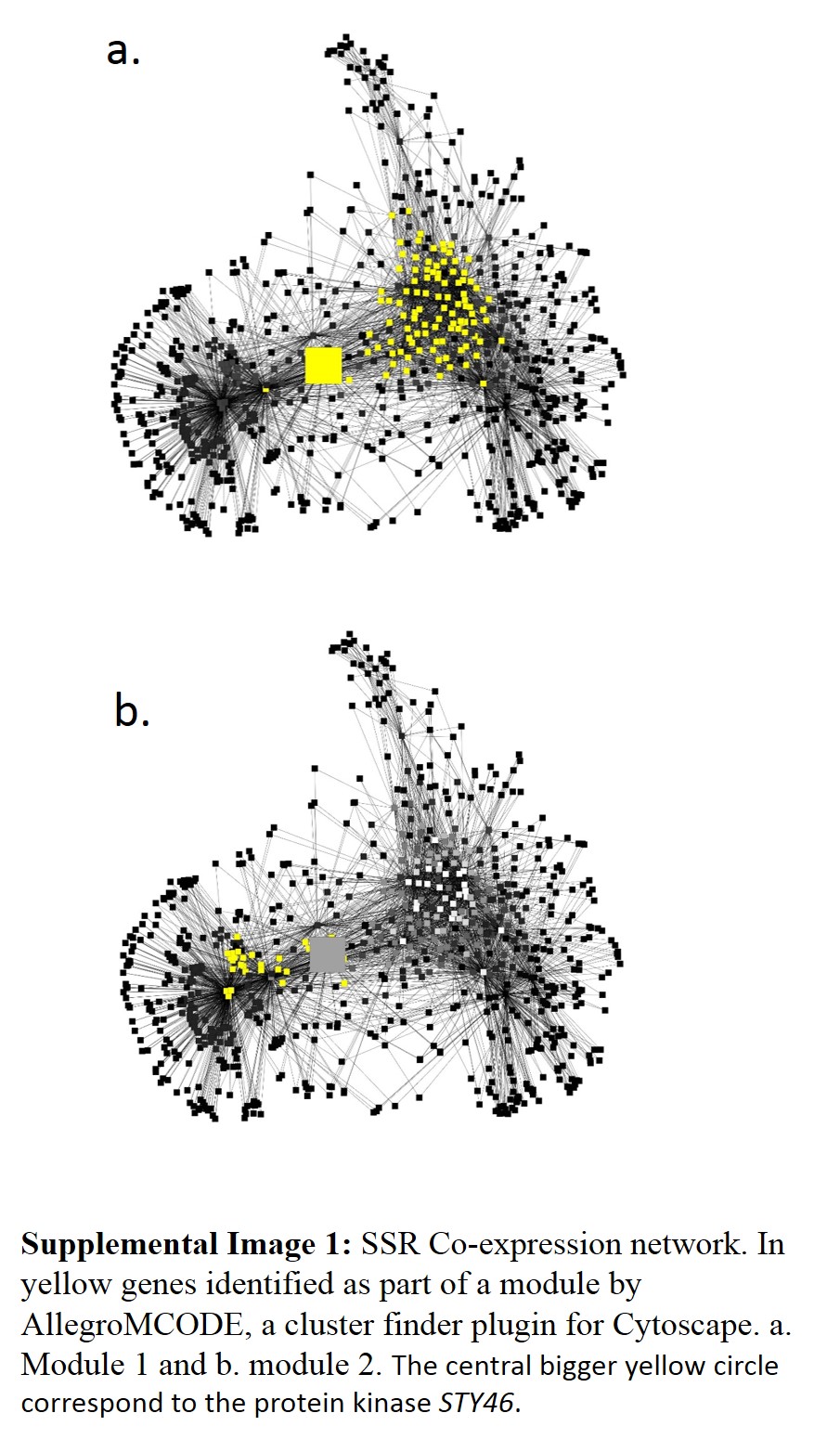

Supplement: Supplementary file 8 [file Image1.JPEG]
